# Walking School Buses as a Form of Active Transportation for Children—A Review of the Evidence

**DOI:** 10.1111/josh.12239

**Published:** 2015-01-21

**Authors:** Liz Smith, Sarah H. Norgate, Tom Cherrett, Nigel Davies, Christopher Winstanley, Mike Harding

**Affiliations:** ^1^ Psychology and Public Health, School of Health Sciences, Allerton BuildingUniversity of SalfordGreater ManchesterM6 6PUUK; ^2^ Logistics and Transport Planning Transportation Research Group, Engineering and the EnvironmentUniversity of SouthamptonRoom 4057, Building 176, Boldrewood Campus, Burgess RoadSouthampton SO16 7QFUK; ^3^ InfoLab21Lancaster UniversityLancaster LA1 4WAUK; ^4^ Associate InfoLab21Lancaster UniversityLancaster LA1 4WAUK

**Keywords:** walking school bus, active transportation, interventions, time, smart mobility

## Abstract

**BACKGROUND:**

Walking school buses (WSBs) offer a potentially healthier way for children to get to school while reducing traffic congestion. A number of pressing societal challenges make it timely to evaluate evidence of their value.

**METHODS:**

Studies that focused solely on WSBs were identified through online and manual literature searches. Twelve WSB studies involving a total of 9169 children were reviewed. Study aims, designs, methods, outcomes, and barriers and facilitators were examined.

**RESULTS:**

WSBs were found to be associated with increased prevalence of walking to school and general activity levels although not always significantly. Time constraints emerged as barriers to WSBs, impacting on recruitment of volunteers and children to the WSBs. Facilitators of WSBs included children enjoying socializing and interacting with the environment.

**CONCLUSIONS:**

Preliminary evidence of the health value of WSBs was demonstrated, along with recommendations for the design of future studies. By tackling barriers of time constraints, volunteer recruitment, and parents' safety concerns while at the same time, increasing convenience and time savings for families, future WSBs are likely to be more sustainable and taken up by more schools. Implications for future innovation in school health were identified.

Walking school buses (WSBs) are commonly cited as one form of active transportation (AT) used on the school commute in the United States, Canada, Europe, Australia, and New Zealand.^1^, ^2^, ^3^, ^4^, ^5^, ^6^, ^7^, ^8^, ^9^, ^10^, ^11^, ^12^ WSBs can be as informal as a small number of families taking it in turns to walk a group of children to school. However, those reported in the literature are predominantly highly structured, involving specific routes, timetables, and trained volunteers. Similarly to other forms of AT, WSBs could increase physical activity in children potentially impacting health while simultaneously reducing congestion outside school gates, but to date, there has been a lack of evidence to quantify this.

Trends from North America, Europe, and Australia have shown consistent declines in the proportions of pupils walking to school and significant increases in proportions being driven. In the United States, the number of children being driven to school has been increasing since 1969 with a decline in walking.^13^ In Britain, the number of 5‐ to 10‐year‐olds being driven to school rose slightly from 38% in 1995/1997 to 42% in 2009^14^ with similar findings reported in New Zealand.^15^, ^16^ Research in Canada reported that only 30% of elementary school pupils in Montreal and Trois‐Rivières reached school on foot or by bicycle.^17^ In Australia, it was reported that only 39% of a self‐selected group of respondents of a primary school in Brisbane ever walked to school,^18^ with similar findings reported for a Sydney school.^19^ The decline in walking to Britain's schools is strongly associated with affordability of cars, which increased during a period of rapid economic growth between 1980 and 2005.^20^ The greenhouse gas footprint for English schools in 2006 was estimated at 9.4 million tons of carbon dioxide equivalent and 16% of that came from school transport.^21^ Walking school buses have the potential to lower these figures.

Although WSBs are not a new phenomenon, the idea originating from Engwicht in 1992,^22^ a number of pressing societal challenges make it timely to revisit their worth. In the United States and the UK, there are concerns about plans to eliminate state funding for school buses which could lead to further children being driven to school in cars.^23^, ^24^ Furthermore, overweight and obesity in children has been rising for several decades in the United States and Canada.^25^ In the United States, childhood obesity rates increased from 5.0% to 10.4% in 2‐ to 5‐year‐olds, between 1976 and 2007‐2008.^26^ In Canada, overweight/obesity rates for 2‐ to 17‐year‐olds increased from 15% to 26% between 1978/1979 and 2004.^27^ In Britain, obesity rates in 10‐ to 11‐year‐olds increased from 17.5% in 2006/2007 to 19.0% in 2010/2011.^28^ If these trends continue, it is predicted that the numbers of overweight children in the United States will almost double by 2030 and associated healthcare costs would double every decade.^29^

To what extent would regular walking through a WSB could potentially increase children's physical activity or motivation for physical activity, potentially impacting on child fitness related health. Although reviews about using AT to get to school exist^30^, ^31^, ^32^ to date, they have tended to focus on AT overall rather than any specific focus on WSBs. In the light of this potential, it is timely to review the evidence base around WSBs, and in particular to examine whether there is any robust evidence of WSBs for children's fitness‐related health or related factors. Alongside this, to increase knowledge about the rigor of research methods used to investigate the role of WSBs in children's fitness related health, with a view to making recommendations for future studies. In so doing, this research reviews studies that focus solely on WSBs as this has not been done previously.

## METHODS

### Participants

This review involves 12 key studies with 9169 children and 326 schools were studied as well as 1113 WSB coordinators and/or parent volunteers and 78 other key stakeholder informants.

### Data Sources

An in‐depth literature search was conducted using the following electronic databases: Web of Science, BIOSIS Citation Index, BIOSIS Previews, Medline, CINAHL, Cochrane Library, Psychinfo, SPORTDiscus, Sociological abstracts, and ERIC. Three groups of search descriptors were used including (1) WSB, (2) active child transportation, and (3) active child travel. The electronic search was supplemented by reviewing relevant bibliographies.

### Procedure

Three inclusion criteria were used to identify review studies: (1) studies focused solely on WSBs and did not compare WSBs with other AT methods such as cycling; (2) study settings were anywhere but papers were written in English; and (3) studies were published in peer‐reviewed journals. The online literature search produced 147 hits. Abstracts were scanned and where inclusion criteria were met, full papers found. Excluded papers comprised studies comparing WSBs with other forms of AT, conference talks, or posters (N = 136). A review of articles' reference lists elicited 1 additional study, increasing the number of review studies to 12.

### Data Analysis

Although 3 of the 12 identified studies were carried out by the same authors and colleagues,^8^, ^9^, ^10^ only the 2011 and 2012 study shared the same participants. Although other authors^1^, ^2^, ^4^, ^5^, ^6^ carried out several studies, they were reported as independent studies. The 12 studies were reviewed and the following data extracted: (1) when study was carried out and for how long; (2) where the research was carried out and any settings/sociodemographic details; (3) aims of studies; (4) design or methods used; (5) number of schools/participants used; (6) outcomes of the studies; (7) facilitators of WSBs; and (8) barriers to WSBs.

## RESULTS

### Study Settings

Walking school buses studies were carried out using samples from Australia, New Zealand, and the United States with a variety of sociodemographics between 2001 and 2012. For example, one study was carried out as a case study at just 1 school that the authors described as “resource rich” and “research informed” in New Zealand. 4 In contrast, 3 studies were carried out in the United States with low‐income public elementary schools that were socio‐economically disadvantaged.^8^, ^9^, ^10^

### Study Aims and Methods Used

Two different categories of studies emerged from the review. Table 1 presents 6 studies having the common purpose of evaluating the impact of WSBs on children's activity, health, and safety awareness. Table 2 summarizes 6 studies aiming to contribute to the knowledge around the developmental aspects of WSBs including barriers and facilitators. One study had both purposes but was included with category 2 developmental studies as it was a feasibility study.^7^ Overall, studies used various methods, for example, one of the category 1 studies aimed to test specific hypotheses that WSB programs would increase children's active commuting to school and their daily activity levels used a cluster randomized controlled trial (RCT).^9^ In contrast, one study from category 2 aimed to add to the understanding of the development of WSBs in the region and successes and challenges^2^ used a longitudinal design with surveys.

**Table 1:** Aims and Methods for Studies of WSB Impact (Category 1)

Study	Study Length	N (I/C)	Study Aim	Method of Evaluation	Robustness of Evidence
Heelan et al^3^	2 years	324 (201/123)	To evaluate school‐wide prevalence of walking to school and to compare activity levels among 2 WSB intervention schools and 1 control school.	A quasi‐experimental design was used with 2 intervention schools and 1 control school in Nebraska, USA. WSBs had designated stops within a 1‐mile radius of schools and adult leaders. This study evaluated school‐wide prevalence of walking to school by self‐report at 6 time points. This study also compared objective physical activity levels among a subsample of research participants (201 intervention children and 123 control children) by having participants wear an accelerometer during 4 time periods to assess daily physical activity. They were also measured for body mass index (BMI) and body fat 2 times a year.	Prevalence of walking to school was measured by self‐report which could have limited validity of the study. However, authors measured objective physical activity using accelerometer but only among a subsample of children at 4 time periods.
Mendoza et al^8^	1 year	820 (347/293)	To evaluate the impact of a WSB program on student transport in a low‐income, urban neighborhood, specifically, would a WSB program increase the proportion of students walking to school and decrease the proportion being driven in short‐ and long‐term.	Controlled, quasi‐experimental trial with cross‐sectional assessments. There was 1 intervention school with a WSB program with parent volunteers and a part‐time coordinator and 2 control schools. All schools were urban, socioeconomically disadvantaged, elementary schools in Seattle, WA, USA. This study assessed students' method of transportation to school by a classroom survey at baseline and 1‐year follow‐up. McNemar's test was used to examine the change from baseline to 12‐month follow‐up for walking versus all other forms of school transport at the intervention or control schools.	This study assessed travel to school method self‐report publicly in classrooms which could limit validity. Also schools were not randomized but authors propose that control schools were comparable to intervention school (in that they all served predominantly disadvantaged, minority populations) which should minimize threats to internal validity.
Mendoza et al^9^	5 weeks	149 (70/79)	To evaluate the impact of a WSB program on children's rates of active commuting to school and on daily moderate‐to‐vigorous physical activity (MVPA).	Pilot cluster randomized control trial with 4 intervention and 4 control schools (all low‐income public elementary schools in Houston, TX, USA). Intervention schools had 1‐3 WSBs with trained staff, to and from schools, 5 days per week. Outcomes were measured the week before intervention (time 1) and during weeks 4 and 5 of intervention (time 2). The main outcome (percentage of trips made by active commuting) was assessed every school day for 1 week during times 1 and 2 using a questionnaire which authors report had a high test‐retest reliability and convergent validity with parental report. This asked children how they got to school. Children had to select 1 answer (from: school bus, carpool, car, metro bus, walked with an adult, walked without an adult, or biked). Walking or biking was considered active commuting. The secondary outcome (MVPA minutes per day) was measured using GT1M accelerometers worn by students for 7 days at times 1 and 2. These measured acceleration in the vertical plane and intensity every minute. A valid day was defined as 10 hours (600 minutes) of accelerometer wear. Participants who had at least 1 valid day were included in analyses.	A brief intervention period, therefore, limited generalizability. There were also baseline differences in percentage of active commuting (intervention 23.8%, control children 40.2%). Al though they used self‐report to measure active commuting, authors say study had good convergent validity as this was checked with parental report.
Mendoza et al^10^	5 weeks	149 (70/79)	To test feasibility of evaluating changes to pedestrian safety behaviors (PSB) during a WSB program. Outcomes measures were pedestrian safety behaviors related to crossing a street at a school level prior to WSB and during WSB trial.	This observational study was a pilot study of children's pedestrian safety behaviors and was conducted as part of the cluster RCT of WSB program (Mendoza et al^9^). Children's pedestrian safety behaviors related to crossing a street were observed by trained research assistants using a previously validated observational instrument. Children of all grades were unobtrusively observed at major intersections. Each of these behaviors ([1] crossed at a corner or crosswalk, [2] crossed with an adult or safety patrol, [3] stopped at the curb, [4] looked left‐ right‐left, and [5] walked and did not run across the street) were scored (yes = 1; no = 0) and a composite score (the sum from 0 to 5 of the 5 behaviors) were determined for each school.	This study used cross‐sectional school‐level data of pedestrian behavior of children of any grade and not longitudinal data on just the fourth graders who were in the WSB study. This could have diluted the impact of the intervention because many of the observed children at intervention schools did not use the WSB program. However, this enabled “real world” observations.
Moodie et al^11^	1 year	7840 (modeled)	To assess from a societal perspective the effects on BMI and disability adjusted life years (DALYs) of the Victorian WSB program if applied throughout Australia. Cost offsets and DALY benefits were modeled until the eligible cohort reached 100 years of age or death.	This study used a logic pathway to model the effects on BMI and DALYs of the Victorian WSB program if applied throughout Australia. Cost offsets and DALY benefits were modeled until the eligible cohort reached 100 years of age or death. The reference year was 2001. Second stage filter criteria (“equity,” “strength of evidence,” “acceptability,” “feasibility,” “sustainability,” and “side‐effects”) were assessed to incorporate additional factors that impact on resource allocation decisions.	Reports of missing data for 7 of the 33 local governments with WSB Programs therefore program activity may have been underestimated. Evaluation assumed the intervention to be in steady state (ie, implemented and working at its full effectiveness), but authors found unutilized capacity. The authors report that the number of participating local governments, schools, WSBs per school, and children per WSB could all be increased without expansion of infrastructure capacity at national, state, local government, or school level.
Sirard et al^12^	1 week	11 (5/6)	To (1) test the feasibility of a WSB as an intervention strategy to increase children's physical activity and (2) to identify any changes in physical activity from walking to school.	A randomized, controlled trial design. All participants attended an elementary school in Menlo Park, CA, USA and completed 1 week of baseline automobile commuting. After randomization, control group continued to be driven to school and intervention group used the WSB (route = 1.1 km). Inclusion criteria were that participants had to be in third‐fifth grade and were driven to school 4 or more days per week. Twelve students were randomized to control (N = 6) or intervention (N = 6) group but 1 had monitor malfunction, leaving only 5 in the intervention group. Students were instructed to wear Acti‐Graph monitors for 14 consecutive days except when swimming, bathing, or sleeping and to maintain normal activities during baseline week. Accelerometer data were summarized for total week and weekdays. Each weekday was also divided into 4 time blocks (before, during, afterschool, evening). Average monitor counts per minute and the average percentage of time spent in moderate‐to vigorous physical activity (%MVPA) were calculated for each student.	This was a very small study with only 5 students in the intervention group and 6 in the control group. Also this study was carried out over a very short time span.

N, number of children; WSB, walking school bus; RCT, randomized controlled trial.

**Table 2:** Aims and Methods for Studies of Developmental Aspects of WSBs (Category 2)

Study	Study Length	N	Main Study Aim	Method of Evaluation	Robustness of Evidence
Collins and Kearns^1^	2 months	45	To gather information about WSBs operating throughout Auckland, New Zealand, to create a regional “snapshot” of WSBs in Auckland.	A telephone survey design based on a question schedule in consultation with transport and road safety officials. Forty‐five interviews with 23 school representatives (mostly principals) and 22 WSB coordinators each of 25‐40 minutes duration were carried out providing information on 29 of the 34 schools with WSBs. Descriptive statistics and thematic analysis were carried out on the data.	Cross‐sectional view of benefits and challenges of WSB schemes from the perspective of 2 stakeholder groups (school principals and WSB coordinators).
Collins and Kearns^2^	5 years	998	To understand the development, changes, challenges, and successes of WSBs in Auckland, New Zealand.	This research was a longitudinal assessment of WSBs using surveys which varied slightly from year‐to‐year—reflecting evolving knowledge and interests of researchers and the Auckland Regional Transport Authority (ARTA). In 2002, telephone interviews were used to collect data form principals of all Auckland primary schools with WSBs and also parent coordinators of each route. In 2003, this was repeated but with only parent coordinators. In 2004, 2005, and 2006, self‐completion questionnaires were sent to parent coordinators of all known WSBs (and response was linked to a $200 ARTA grant, organized independent of researchers). Questions remained largely unchanged. Average response rate was 66% and highest was 79% in the 2006 survey.	While this study has a large sample and good response rates, caution should be taken in generalizing results outside of Auckland, New Zealand.
Kearns et al^4^	1 month	23	To examine parents' perceptions of benefits, limitations, and long‐term viability of WSBs, to understand the experience of walking the WSB, and to uncover and understand child pedestrian safety issues and attitudes to the WSB held by representatives of stakeholder groups.	This was a mixed‐method evaluation of one school's WSB. First, parents who entrusted their children to the WSB, or who were “drivers,” were surveyed on their perceptions of its benefits, limitations, and long‐term viability. This method yielded a 44% response rate (16 of 36 involved families). Second, one author observed and conversed with volunteer drivers and child passengers of the WSB for 1 week to understand the experience of walking with the WSB. Third, to try and understand child pedestrian safety issues and attitudes to the, another author conducted interviews with the school principal, the school's road safety coordinator, the retired volunteer “driver,” parent coordinator, chair of “Safe Journeys Coalition,” Auckland City Council's Road Safety Advisor, and a representative of “Accident Compensation Corporation.”	A mixed method approach focused on one school in Auckland, New Zealand.
Kingham and Ussher^5^	Duration not stated but research carried out mid‐2003	55	This study aimed to examine the long‐term success and durability of WSBs in Christchurch, New Zealand, and to determine factors which contribute to the growth and longevity of WSBs as the authors point out that many evaluations occur soon after WSBs have been set up therefore missing long‐term successes.	This study used a combination of interviews and questionnaires with people involved in running the WSB. In total, 33 interviews were carried out with current or past WSB coordinators from 11 schools, council officials responsible for WSBs, and school principals where WSBs operated. Questionnaires were also sent to all the remaining Christchurch primary school principals to find out why WSBs had not been established at their school. Council data were also examined for start dates, numbers involved in WSBs, and any evaluations that had been carried out.	This study describes in depth the factors contributing to success, growth, and durability of WSBs in Christchurch, New Zealand. The response rate for questionnaires is not reported.
Kingham and Ussher^6^	Not stated	33*	This study aimed to identify the perceived benefits of WSBs with a particular focus on the impacts on children's independent mobility, by interviewing people involved in the day to running of the scheme in Christchurch, New Zealand.	This study used a combination of interviews and questionnaires with people involved in running the WSB. In total, 33 interviews were carried out with current or past WSB coordinators from 11 schools, council officials responsible for WSBs, and school principals where WSBs operated. Questionnaires were also sent to all the remaining Christchurch primary school principals to find out why WSBs had not been established at their school. Council data were also examined for start dates, numbers involved in WSBs and any evaluations that had been carried out.	This study reports the perceived benefits of WSBs. The response rate for questionnaires is not given. This study may also have used the same sample as the above study, but there was no citing of the 2005 paper in the 2007 paper.
Kong et al^7^	10 weeks	34	To examine the feasibility of implementing a WSB and to present the lessons during the implementation of a modified WSB program in a predominantly Hispanic elementary school in Albuquerque, New Mexico.	This feasibility trial used a process evaluation of 2 WSBs at 1 elementary school in Albuquerque. Twenty‐nine participants (kindergarten‐5th grade students living within a 1‐mile radius of school) had a physical examination and BMI percentiles taken at beginning and end of trial. A part‐time WSB coordinator, a lead parent volunteer, and 9 volunteer parents ran the WSBs along police approved designated routes. Four health themes were given to participants to try and they were asked to discuss strategies for implementing then on WSB to and from school. Qualitative and quantitative data were obtained from coordinator field notes, attendance records, students' and parents' satisfaction surveys, focus group with adult volunteers, and interviews with 2 lead coordinators.	This study used a small convenience sample which may not have been representative. Caution is needed in making generalizations from results.

WSB, walking school bus; BMI, body mass index.

N, number of participants (WSB coordinators, volunteers, parents, children, or key informants). For further breakdown, see Table 3.

#### Category 1: impact of WSBs

Four studies in category 1 examined the impact of WSBs on children's activity,^3^, ^8^, ^9^, ^12^ with another focusing indirectly on activity as the authors suggested that WSBs offer opportunities for children to increase activity which together with changes in diet could reduce the likelihood of childhood obesity. The authors of this study aimed to assess the incremental cost effectiveness of a WSB program as an obesity prevention measure^11^ if the WSB program was applied throughout Australia, by employing a logic pathway to model the effects on body mass index (BMI) and disability adjusted life years (DALYs). The sixth study in this category aimed to assess the feasibility of evaluating changes to pedestrian safety behaviors (PSBs) during a WSB program.^10^ Two studies used quasi‐experimental designs, where no random assignment was made, but data were collected through repeated measurements over time. One study compared activity levels using accelerometers and prevalence of walking to school among 2 WSB intervention schools and 1 control school at 6 time points over 2 years.^3^ The other study tested whether a WSB program would increase the proportion of students walking to school and decrease the proportion being driven.^8^ This was tested at 1 intervention and 2 control schools at 4 time points over 12 months. However, both studies^3^, ^8^ used self‐report for measuring prevalence of walking to school which could have limited the validity of their findings.

Three studies used RCTs which are often considered to be reliable and “gold” standard methodology for testing efficacy of interventions, as RCTs reduce false causality and bias.^33^ The first study involved used an RCT to test how feasible it was to use a WSB as an intervention strategy to increase children's physical activity and to identify any changes in physical activity from walking to school.^12^ All participants came from 1 school and completed a baseline week of automobile commuting. In the second week, they were randomized to either control (N = 6 continued to be driven to school) or intervention conditions (N = 5 used the WSB) when physical activity was monitored using accelerometers. In a second study, authors used a cluster RCT among 4th graders from 8 schools, 9 where schools rather than individuals were randomly assigned to intervention (N = 4) or control conditions (N = 4). Intervention schools had 1‐3 WSBs each day for 5 weeks. Weekly rates of active commuting was monitored again by self‐report and moderate‐to‐vigorous physical activity (MVPA) was measured using accelerometers before and during week 4‐5 of the intervention and were compared for intervention and control groups. Another study by the same authors, conducted as part of the above study, assessed the feasibility of evaluating changes to PSBs during the WSB program by unobtrusively observing children of all grades at major intersections at each school.^10^

#### Category 2: developmental aspects of WSBs

Of the 6 studies in category 2, Table 2 shows that 3 involved use of interviews and surveys/questionnaires, with one aiming to identify factors contributing to growth and longevity of some WSBs in contrast to others which declined or stopped.^5^ The other 2 studies examined benefits of WSBs, 4, 6 with one investigating the potential of WSBs to promote well‐being and safe use of urban streets by children^4^ and the other examining WSBs' less quantifiable perceived benefits of WSBCs, council officials, and school principals.^6^ Another study involved 2 WSBs for 10 weeks accompanied by use of student/parent satisfaction surveys, field notes, registers, and a focus group to evaluate feasibility.^7^ Telephone surveys were completed by 29 of the 34 schools with WSBs to create a regional “snapshot” of WSBs in Auckland, New Zealand.^1^ Another study used a longitudinal design with 5 annual surveys with principals and parent coordinators to try and understand the development of WSBs in Auckland, New Zealand.^2^

The ages of the 9169 children discussed in the reviewed studies ranged from 5 to 11. Table 3 shows that 5 studies used key informants^1^, ^2^, ^4^, ^5^, ^6^ but 2 did not report how many.^2^, ^6^ All authors describe the WSBs used in their studies but WSB stops or pickup points were only described in 1 of the 12 studies. Here, WSBs had between 1 and 3 specified pickup points along their routes and the shortest WSB also briefly went door‐to‐door to pick up some students in a neighborhood housing project.^8^ Six other studies had stops which authors only mentioned in passing,^3^, ^4^, ^7^, ^9^, ^10^, ^11^ eg, parent volunteers reported being unsure of how to proceed in the absence of expected adults or children at pickup points.^7^ Four studies made no mention of stops or pickups^2^, ^5^, ^6^, ^12^ and 1 study only mentioned them in their introductory description of WSBs in general.^1^ Findings of studies in category 1 were mixed results partly due to diverse outcome measures being used including the prevalence of walking and active commuting,^3^, ^8^, ^9^ physical activity/MVPA,^3^, ^9^, ^12^ percentage of body fat,^3^ street crossing behaviors,^10^ BMI, and DALYs saved.^11^

**Table 3:** Details of Participants and Overview of Study Outcomes

Study	Schools	Children	WSBCs and Parents	Other Key Informants	Study Outcomes
Collins and Kearns^1^	29	0	22	23	WSBs were highly concentrated in low‐deprivation neighborhoods. Participants identified benefits from WSBs, eg, an estimated 429 saved vehicle journeys each day, parent's not having to drive children to school and navigate traffic, and parents' peace of mind that children would get to school safely. However, they conclude that WSBs have limited ability to address public health challenges in an inequitable and car‐dominated urban political system.
Collins and Kearns^2^	66	0	998	No report	The number of WSB routes was continuing to grow, but most activity still remained in the wealthiest areas. Sense of community, opportunity for exercise and health promotion, reduction in car use and local congestion, and reduced risk of injury risk for child pedestrians were all reported as benefits of WSBs.
Kearns et al^4^	1	No report	16	7	Health benefits of walking, giving a sense of purpose, talking, telling jokes, independence, parents' time saved, removal of the “hassle” of driving, and knowing children were safe were all reported as benefits, although these authors conclude that WSBs are an ambivalent response to the hegemony of motorized transport.
Kingham and Ussher^5^	11	0	33	22	WSBs are suffering a significant decline. Difficulties included a lack of volunteers, lack of children through them wanting to make the journey alone, insufficient ongoing support from the school or council.
Kingham and Ussher^6^, *	11	0	33	No report	Social connections, promotion of community spirit, enhancement of children's health, time saved parents not having to take their children to school every day, getting children into the habit of walking, enjoyment from socializing and what they saw, and children increasing their independence were also reported as benefits.
Kong et al^7^	1	25	9	0	Student and adult participants reported high levels of satisfaction with the WSB. They reported that the WSB provided a supportive and safe environment to promote social interaction and physical activity, although no significant differences in BMI were found between pre‐ and post‐intervention times. WSB studies in urban, underserved school districts are feasible but require attention to ensure participants' involvement, safety, and investment from stakeholders.
Heelan et al^3^	3	324	0	0	At baseline, similar modes of transportation used by children attending WSB and control schools. However, at each later time point, a significantly greater percentage of children actively commuted to and from WSB schools compared with control school. On average, all post‐baseline results over the 2 years, 36.2% of children from WSB schools actively commuted at least 50% of the time compared with 26.2% of control school. Also, frequent walkers across all schools did 25% more physical activity and gained 58% less body fat compared with passive commuters.
Mendoza et al^8^	3	820	0	0	Although no significant differences between proportions of students walking to school at intervention and control schools at baseline, significantly higher proportions of students walked to school at the intervention school than control schools by 1 month into the intervention and remained so by 6‐ and 12‐month follow‐up.
Mendoza et al^9^	8	149	0	0	Intervention schools increased active commuting (AT) from time 1 to time 2, whereas control schools decreased AT. Intervention children increased the daily MVPA from 46 to 48 minutes, whereas control children decreased MVPA from 46 to 41 minutes. The WSB children achieved 7 minutes/day more MVPA than control children.
Mendoza et al^10^, †	As row above	As row above	As row above	As row above	The WSB was associated with more children crossing at an intersection, but fewer children fully stopping at the curb. They speculated that the latter result may have been confounded as children on WSB were directed to cross by an adult. They recommend it is feasible to collect pedestrian safety behaviors and changes to them during WSB studies.
Moodie et al^11^	192	7840 (modeled)	0	26	No increase in the number of children walking due to WSBs. The WSB program was not judged as an effective or cost effective measure to reduce childhood obesity. This was partly because WSB has not yet reached its “steady state,” ie, still room for expansion within current infrastructure arrangements. There were other potential benefits.
Sirard et al^12^	1	11	0	0	Intervention children significantly increased their physical activity and percentage of MVPA before school compared to control school children. This difference was even greater during general commute time (45 minutes before school) where there was no change for controls but intervention children added an average of 14 minutes of MVPA. However, no significant group differences found for total daily or weekday physical activity. Five of the 6 students in the intervention group said they would continue to walk to school.
Total	326	9169	1111	78	

WSB, walking school bus; BMI, body mass index; MVPA, moderate‐to‐vigorous physical activity.

* This could have been same sample as Kingham and Ussher^5^ study.

† This was the same sample as Mendoza et al.^9^

#### Outcomes from studies in category 1: impact of WSBs

In all 3 studies where researchers examined the prevalence of walking to school, the consensus was that compared with control schools, interventions increased active commuting at each time point after intervention had started.^3^, ^8^, ^9^ MVPA also increased, although not significantly. For instance, one study's intervention children increased daily MVPA from 46 to 48 minutes, whereas control children decreased MVPA from 46 to 41 minutes.^9^ Another study's intervention children added an average of 14 minutes of MVPA during general commute time (45 minutes before school), but no significant group differences were found for total daily activity.^12^ A third study found that frequent walkers across all schools did 25% more physical activity compared with passive commuters and gained less weight and BMI units over the 2 years^3^ but some were from control schools.^3^ Authors from another study judged the WSB program as not an effective or cost effective measure for reducing childhood obesity,^11^ although it was suggested that this was partly because WSBs had not yet reached their “steady state” and still had room for expansion.^11^ The final study in this category found mixed results for the impact of WSB programs on PSBs.^10^

#### Outcomes from studies in category 2: developmental aspects of WSBs

Category 2 studies also had mixed results.^1^, ^2^, ^4^, ^5^, ^6^, ^7^ In one study, students and adults reported high levels of satisfaction with the WSB^7^ and that it provided a supportive and safe environment to promote social interaction and physical activity. In this study, 5 of 9 parent volunteers rated that the WSB increased their children's walking “a lot” and 4 “somewhat” and all 22 students surveyed believed they were walking more,^7^ but no significant differences in BMI were found between pre‐ and post‐intervention times. In another study, authors concluded that WSBs only have limited ability to address public health challenges in an inequitable and car‐dominated urban political system.^1^ One study found that WSB routes to be growing but activity remained mostly in wealthiest areas,^2^ whereas another found that routes were in decline^5^ and talked about barriers and challenges.^5^ Enjoyment of health benefits,^2^, ^4^, ^6^ community spirit,^2^, ^6^ socializing,^4^, ^6^ increased child independence,^6^ and parents' time savings^4^ were also reported as benefits and facilitators.

### Facilitators of WSBs

#### Enjoyment on use of WSBs

Six studies reported on the enjoyment of children and parents participating in WSBs, particularly through socializing with friends and getting to know people of different ages.^2^, ^7^, ^12^ Observations also revealed that children enjoyed talking and telling jokes on the WSB.^4^ Social connections were built through children's and adults' involvement with the WSB,^2^, ^7^ and WSBs were reported to help build a sense of community.^2^, ^6^ One study recommended emphasizing these social benefits to support volunteer/child recruitment.^5^ Some children began to prefer walking to being driven,^6^ their enjoyment coming from not only socializing but also from what they saw and did on the WSB. Examples included finding creatures, walking in snow, feeding ducks, and so on. Kingham and Ussher point out that this interaction with the environment, which children are deprived of when driven, allows them to enjoy the childhood adventures that other researchers^34^, ^35^ have stressed as being important for children's cognitive development and academic performance.^36^ Children also enjoyed more independence as parents only accompanied the WSB once a week.^6^ Children and adults also enjoyed health and fitness benefits of WSBs^2^, ^4^, ^6^ and the small but symbolically important incentives given to loyal walkers,^2^, ^4^, ^5^, ^7^ including recognition,^2^ Frisbees,^7^ “Zippy the Zebra” toys,^4^ and personal/house points.^5^

#### Time saving

Parents' time savings were reported in 5 studies as major benefits of WSBs. Almost 50% parent coordinators interviewed discussed time savings in one study, as they did not have to escort their children to school every day.^6^ Some parents felt “better off” as they had more time to themselves and had relieved the stress of driving and finding parking spaces.^1^, ^4^ One parent thought it was great that on 4 days she just had to get her children out of the door and they would get to school safely.^6^ Another participant reported that the WSB enabled her to work part‐time and when she did not work she “conducted” the WSB.^6^ These authors suggest that this finding supports previous unpublished research which stressed the importance of parents' time savings, some of whom originally expected involvement with WSBs to be time consuming. Another study alluded to time savings through reduced levels of parental chauffeuring.^2^ One study proposed that WSB structures could help address the barrier of lack of time to WSBs.^3^

#### Information provision and promotion of WSBs

Five of the 12 studies reviewed discussed information provision and promotion of WSBs, whereas elsewhere it has been reported that many studies do not describe “marketing” of interventions or information for users, which if used could positively impact on uptake.^32^ Three studies disseminated WSB information in school newsletters.^5^, ^7^, ^8^ Meetings and workshops were also a common method of disseminating information about WSBs.^2^, ^5^, ^8^ Christchurch City Council held just one WSB meeting and then intentionally left parents to their own devices to gather more information to encourage them to take ownership of it.^5^ One WSB was promoted through its symbol of a Zebra, derived from Zebra crossings being safe places to cross.^4^ This symbol was posted on signs, walls, and telegraph poles and identified where children were to meet the WSB. It gave the WSB an identity, advertised it, and attracted people's attention resulting in parents asking for information about enrolling their children.^4^

### Barriers to WSBs

#### Safety concerns

Road safety concerns emerged as the most common barriers to WSBs and were discussed in 10 of 12 studies.^1^, ^2^, ^3^, ^4^, ^5^, ^7^, ^9^, ^10^, ^11^, ^12^ One study had police walk the route with parents to discuss safety, trained volunteers, and paid crossing guards to stay longer for the WSB but road safety concerns still emerged.^7^ Thirty‐three percent of parents interviewed and 10 coordinators had serious concerns about children's safety on WSBs, especially on routes crossing main roads with heavy traffic.^5^ Walking school bus coordinators reported the dominance of cars, cars not stopping, and extremely busy roads with 70 km/h speed limits.^2^ It was suggested that routine walking with WSBs may generate knowledge of the community and safety benefits.^2^ One study set out to test the feasibility of evaluating changes to PSBs during a WSB program but found mixed results partly because one outcome measure, “stopping at the curb,” was confounded by WSB coordinators directing children to cross.^10^ Other studies suggest that if WSBs attract enough children, they could result in safer traffic environments around schools, reduce congestion, accidents, and pollution^11^and may be able to address parents' concerns for safety.^4^, ^9^ One school principle in Auckland, concerned about safety, gave the responsibility of the WSB to teachers as they were not confident of parents taking on the responsibility of the children's safety.^1^

#### Recruitment

Recruitment of volunteers and children for WSBs was a commonly reported challenge. Complex family travel schedules and lack of motivation for walking in winter months hampered WSBs.^1^ It was proposed that even “successful” WSBs should recruit new volunteers and users regularly as families leave neighborhoods, adopt new schedules, and older children leave the WSB.^1^ Many parents were keener for their children to use the WSB than they were to volunteer.^2^ One study could only operate its WSB once or twice a week because of limited volunteer availability.^8^ Another study reported unanticipated interest from children resulting in the creation of a second unplanned WSB which was inadequately staffed.^7^ Particular difficulties were reported in getting male parent volunteers.^4^ It was recommended that to address reported recruitment problems, schools/councils should ensure that volunteers feel valued^5^ perhaps by publicizing research showing children with parents involved with their school performing better academically than children from more isolated families.^37^ Only 50% of children registered to use the WSB actually use it on any given morning.^2^ Outcomes from 2 studies demonstrated that as children developed their skills and confidence through WSBs, they then preferred to walk unescorted usually when they were around 9–10 years old, and WSBs collapse through lack of children.^5^, ^6^ One study that found payment for WSB volunteers to be very costly, authors suggest it was so because WSBs still had room for expansion in numbers of participating local governments, schools, and children per WSB.^11^

#### Time constraints

Time constraints were reported in 7 studies and were sometimes cited as the reason behind recruitment difficulties.^7^ Successful and sustainable WSBs need coordinators with time to dedicate to them.^1^, ^8^ School staff's time commitments and parents' work commitments and care for younger children left little time to dedicate to WSBs.^1^, ^2^, ^11^ Some parents' failure to inform WSB coordinators of children's absences led to volunteers feeling unsure of how to proceed and wasted time.^1^, ^2^ Time constraints prevented some families from participating as WSBs required more time than driving children to school and children may have had to change their morning routine, with less time for sleeping and eating breakfast.^12^ One study found that WSBs clustered within less deprived areas and suggested that parents in lower socioeconomic areas often lacked time to volunteer for WSBs.^1^ A few parents put a lot of time into the WSB while others treated it as a babysitting service.^1^ Similarly, another study reported a “free loader” problem, where some parents were keener for their child to use the WSB, than they were to volunteer their time to assist it.^2^ Some motivated volunteers eventually became complacent as it took such a long time to set up the WSB.^1^ It was suggested that the large time commitments needed may not be acceptable to all schools^11^ and that schools/councils should invest in encouraging more people to dedicate time to WSBs. Walking school bus coordinators reported time pressures and suggested that rosters should be flexible to suit family situations.^5^

## DISCUSSION

This is the first review that we are aware of that focuses solely on WSBs and not AT overall and is timely in light of the lack of knowledge about the potential health impact of WSBs. The knowledge gained as a result of this review goes some way toward answering these. First, there is some evidence of WSBs being positively associated with increased proportions of children walking to school,^3^, ^8^, ^9^ although this was measured by self‐report which may be subject to social desirability bias. Walking school buses were also associated with increased activity levels in children,^3^, ^9^, ^12^ and this was measured by more objective means by using accelerometers. Although none of studies directly reported on lowering congestion around the school gate or the greenhouse gas footprint associated with schools, 2 studies reported on WSBs resulting in car journeys being saved^1^, ^2^ which provide preliminary data in this area.

The issue of whether or not WSBs together with changes in diet could have an impact on childhood obesity was partially addressed in that several studies examined BMI before and after a WSB program, but none had any data on children's diet. One study found no statistically significant differences in BMI or body fat between intervention and control groups, but over the 2 years of the intervention while children were developing frequent walkers gained less weight and BMI units, compared with passive commuters.^3^ Another study judged WSBs not to be effective or cost effective measures to reduce childhood obesity, although Moodie et al suggest cost effectiveness would be improved if WSBs were more widespread^11^ as they reported WSBs had room for expansion within current infrastructure arrangements. Another study found no significant differences in BMI between pre‐ and post‐intervention times.^7^

There was some evidence that regular walking through a WSB could potentially teach children road safety skills as one study found that WSB schools had a 5‐fold improvement in children crossing at intersections where they can be seen by traffic versus non‐intersection/mid‐block locations.^12^ However, the same study found that fewer WSB children fully stopped at the curb, but the authors suggested that this latter result may have been confounded as children on WSB were directed to cross by the WSB coordinator and so further investigation is needed.

This review demonstrates that WSBs show promise in helping to increase children's physical activity. However, the scope to make systematic comparisons between study outcomes has been limited by diverse approaches to research methods, with only a limited number of studies adopting randomized control design. Overall, in terms of sample size, this ranged from case studies of individual schools^4^ up to over 192 schools in the utilization of statistical modeling techniques.^12^ Similarly, the duration of study have ranged from 1 week^12^ up to 5 years, 2 with other studies not reporting this information. Related, it would aid comparison across study outcomes if authors reported assessment of the measures of the support for WSBs underpinning interventions.

Overall, the research outcomes showed that setting up and maintaining WSBs are challenging. The main barriers are safety concerns of parents, recruitment of people to run the WSBs, and time constraints. Time emerged as a facilitator and a barrier to WSBs as parents saved time when children were using the WSB then lost time if they were involved in the running of the WSB. Other facilitators of WSBs were found to be the sheer enjoyment of children walking and talking on the WSBs on their way to school and information and promotion of WSBs.

We recommend that a WSB champion or coordinator role could be formalized and paid with teaching assistant or equivalent role being paid an extra hour or 2 to run WSBs at the beginning and at the end of the school day. This would help to address barriers of recruitment for WSBs and time constraints of parents getting involved in the running of WSBs. In light of time constraints discussed above as barriers to WSBs and reported in 7 studies, we also suggest that advances in smart mobile technology may be able to help revolutionize WSBs.^38^ Considering how lives have tended to become governed by society's time schedules, with people steering back‐to‐back commitments of work and family, it is not surprising that time constraints emerged as an issue in this review. Although no studies in this review explore the use of smart mobile technology in the running of WSBs, it is suggested that tracking ability in smartphones could potentially offer parents of WSB users new temporal visibility of WSBs which could optimize fluidity across scheduling boundaries between the morning school run and work start times. Parents could track the position of the WSB on their smartphone and visualize the WSB's current position and its predicted arrival time at their pickup point. This could make it more convenient for parents dropping their children off at the designated stop and enhance their time savings found to be a facilitator in these studies. In addition, tracking ability could enable them to see the WSB arriving at school and go some way to allay their safety concerns.

This review of studies focused specifically on WSBs and has demonstrated that WSBs show some promise in increasing children's physical activity levels. In addition, there were reports that children enjoyed using the WSBs through socializing and also from what they saw and did on the WSB. This may imply that they will stay motivated to walk to school.

To conclude, this timely review of the value of WSBs demonstrated preliminary promising evidence for the impact of WSBs on children's health and development including road safety awareness. It was found that most “impact” studies used at least one objective measure. However, there was insufficient scope to make systematic comparisons across study outcomes. Future research could adopt “minimal” threshold methods standards based on outcomes from the review of methods used in 12 studies. Specifically, future studies could use more objective measures in conjunction with measures of self‐report, thereby triangulating the approach to research methods.

The main barriers to sustainable WSBs were found to be the safety concerns of parents, recruitment of people to run the WSBs, and time constraints. However, time also emerged as a facilitator of WSBs, as parents saved time when children used the WSB. These factors are now considered below, in relation to implications for school health.

## IMPLICATIONS FOR SCHOOL HEALTH

Promoting elementary school communities to run WSBs could increase use of active modes of transport used to get to school as well as children's activity levels. Although the obesity epidemic will not be reversed by exercise alone, incremental increases in children's activity levels on the journey to school could be seen as a healthy step to new habit formation, particularly at this influential point in the life span. In the light of this, we propose specific recommendations for future innovation in schools around both WSB use and research.

First, we reduced barriers to WSB provision in elementary schools by treating WSBs as a service to families. To this end, WSB leaders or champions in elementary schools were appointed, paying them to run WSB services aimed at promoting child health‐related fitness. Crucially, elementary school leaders have a pivotal role to play in awareness raising of what is known about the physical health, independent mobility, road safety awareness, social and cognitive benefits of WSBs to children as well as reducing vehicle congestion with a view to enhancing safety around the vicinity of the school.

Second, we created new partnerships between state colleges and elementary schools. Such partnerships would enable the co‐development of research initiatives for WSBs and a “scale up” of the quality of interventions—especially by way of size, duration, and sustainability—thereby increasing understanding of the value of WSBs for children's health and other developmental benefits. In turn, any improvement in the quality of research design in studies involving interventions around WSB would enable academics to identify principles for methodology, and consider devising a set of “threshold” standards.

Finally, to encourage WSB leaders and school communities to adopt innovative smart mobility initiatives^38^ to support their school WSBs service. This will enable families to access WSB services in a more convenient and time‐efficient way. Significantly, such initiatives for WSBs also have the wider potential to make the communication over the school‐home boundary more porous, empowering families to engage more proactively in a range of school community health initiatives.

### Human Subjects Approval Statement

No human subjects were involved in the review process. The work undertaken in this review was part of a wider project receiving ethical approval by the institutional research governance and ethics panel at the University of Salford, Greater Manchester, UK.
